# Heterogenous Subtypes of Late-Life Depression and Their Cognitive Patterns: A Latent Class Analysis

**DOI:** 10.3389/fpsyt.2022.917111

**Published:** 2022-07-06

**Authors:** Li-Qi Wang, Tian-Hong Zhang, Wei Dang, Sha Liu, Zi-Li Fan, Li-Hui Tu, Ming Zhang, Hua-Ning Wang, Nan Zhang, Qin-Ying Ma, Ying Zhang, Hui-Zi Li, Lu-Chun Wang, Yao-Nan Zheng, Huali Wang, Xin Yu

**Affiliations:** ^1^Clinical Research Division, Dementia Care and Research Center, Peking University Institute of Mental Health (Sixth Hospital), Beijing, China; ^2^Beijing Dementia Key Lab, National Clinical Research Center for Mental Disorders (Peking University), NHC Key Laboratory of Mental Health, Beijing, China; ^3^Shanghai Key Laboratory of Psychotic Disorders, Shanghai Mental Health Center, Shanghai Jiao Tong University School of Medicine, Shanghai, China; ^4^Department of Psychiatry, Xi'an Mental Health Center, Xi'an, China; ^5^Department of Psychiatry, First Hospital/First Clinical Medical College of Shanxi Medical University, Taiyuan, China; ^6^Beijing Key Laboratory of Mental Disorders, National Clinical Research Center for Mental Disorders, Beijing Anding Hospital, Capital Medical University, Beijing, China; ^7^Department of Psychiatry, The Third Affiliated Hospital of Sun Yat-sen University, Guangzhou, China; ^8^Department of Psychiatry, Xijing Hospital, The Fourth Military Medical University, Xi'an, China; ^9^Department of Neurology, General Hospital of Tianjin Medical University, Tianjin, China; ^10^Department of Neurology, The First Hospital of Hebei Medical University, Shijiazhuang, China

**Keywords:** depressive disorder, late-life depression, latent class analysis, depression subtypes, cognitive impairments

## Abstract

**Background:**

Late-life depression (LLD), characterized by cognitive deficits, is considered heterogeneous across individuals. Previous studies have identified subtypes with diverse symptom profiles, but their cognitive patterns are unknown. This study aimed to investigate the subtypes of LLD and the cognitive profile of each group.

**Methods:**

In total, 109 depressed older adults were enrolled. We performed latent class analysis using Geriatric Depression Scale items as indicators to generate latent classes. We compared the sociodemographic and clinical characteristics with cognitive functions between groups and conducted regression analysis to investigate the association between class membership and variables with significant differences.

**Results:**

Two classes were identified: the “pessimistic” group was characterized by pessimistic thoughts and the “worried” group with a relatively high prevalence of worry symptoms. The two groups did not differ in sociodemographic characteristics. The “pessimistic” group showed a higher rate of past history of depression and lower age of onset. The “worried” group had more physical comorbidities and a higher rate of past history of anxiety. The “pessimistic” group was more impaired in general cognitive function, executive function, information processing speed, and attention. Lower general and executive functions were associated with the membership in the “pessimistic” group.

**Conclusions:**

Subjects with pessimistic symptoms and subjects with a propensity to worry may form two distinct subtypes of late-life depression with different cognitive profiles. Further, the cognitive evaluation of subjects with pessimistic symptoms is of utmost importance.

## Introduction

Major depressive disorder (MDD) in later life is highly prevalent (1, 2), often misdiagnosed, untreated (3), and may subsequently lead to impairment of daily life function, cognitive decline, and disability (4). Multiple cross-sectional and longitudinal studies have proven that late-life depression (LLD) is strongly associated with neurocognitive impairment. More than half of LLD patients present significant cognitive impairments (5). Impairments in executive function and information processing speed are frequently observed, and deficits in memory, attention, and visuospatial function have also been reported in quite a few studies (6). These neuropsychological deficits, especially executive function, are correlated with impaired daily life function, the course of depressive episodes, and treatment response (7) to antidepressants in LLD subjects. With China being a rapidly aging country, it is of great importance to acquire a more profound and holistic view of late-life depression and underlying cognitive impairments.

However, studies focusing on LLD are also challenged by the heterogeneity of MDD. Fried et al. (8) discovered that subjects who met the criteria of MDD in the Diagnostic and Statistical Manual of Mental Disorders V (DSM-5) reported a tremendous number of different symptom profiles. Divergent risk factors, biomarkers, and treatment responses were also uncovered among different subjects with MDD (9, 10). For LLD, the situation is complicated by the aging process and the perplexing biological mechanism of LLD, including genetics, vascular factors, inflammation, and neurodegeneration (11). Making accurate diagnoses and treatment plans, improving prognoses, and achieving precision medicine all call for the necessity of tackling the heterogeneity of LLD (12).

Researchers have started to delineate the heterogeneous symptom and cognitive impairment profiles by subtyping LLD based on the age of onset, clinical features, and etiology. An abundance of studies have compared the patterns between LLD subjects with late age at onset (LOD) and subjects with early age of onset (EOD), with more hypochondriasis, somatic symptoms, and less suicidal, pessimistic thoughts endorsed by the EOD group (13). The results regarding the cognitive differences between EOD and LOD seemed inconsistent. EOD subjects were reported in some studies to be more impaired in the domains of memory (14) and information processing speed (15), while LOD subjects had more white matter damage (16) and performed worse in executive function (17). On the other hand, some studies found that LOD subjects had poorer memory performance (18), while EOD subjects scored worse in the executive tests (19). In addition, Alexopoulos et al. (20) proposed that “vascular depression” might be viewed as a particular subtype of LLD. Following this hypothesis, researchers have investigated the difference between the vascular and non-vascular groups. Some reported that the vascular group scored worse in tasks evaluating executive function, information processing speed, and working memory (21, 22), although a longitudinal study reported no difference in symptom profiles between the two groups at baseline (23). However, in general, few consensuses have been reached thus far. The artificial division seems arbitrary with the possibility of obscuring the true nature of the heterogeneity of LLD.

Recently latent class analysis (LCA) has been applied to generate data-driven subtypes and depict the heterogeneity of psychiatric diseases, including depressive disorder. Compared to methods such as principal component analysis (PCA) or factor analysis (FA) that classify variables or items (24), LCA is a person-centered approach that classifies subjects based on latent variables, making it more intuitive to uncover the heterogeneity of mental disorders. Such a method has been applied for subtyping MDD in adult participants with informative results (25). Similarly, some studies also conducted LCA within LLD subjects. For instance, Hybels et al. (26, 27) reported three to four latent groups among depressed elderly individuals, differing mainly in severity and specific symptoms, including appetite and weight loss, thoughts of death, and suicidal thoughts. Veltman et al. (28) found three classes: a moderate-severe class, a severe melancholic class, and a severe atypical class. Pérez-Belmonte et al. (29) also discovered three groups among older subjects with clinical depression: psychosomatic, melancholic, and anhedonic. Subtypes of LLD based on symptomatology facilitate application in clinical practice, but few studies (30) looked deeper and investigated the cognitive difference among latent groups. We expect that investigating the cognitive profile of each subgroup will provide us with a more comprehensive understanding of the heterogeneity of LLD. Additionally, the scales and evaluation tools used in previous studies were designed for the general population with MDD. We presume that using special tools for older adults may be more pertinent for LLD studies.

Therefore, the objectives of this study are as follows: (1) identify the subgroups of LLD using latent class analysis; (2) examine the differences in demographic and clinical characteristics and cognitive function between groups and investigate the associations between class membership and these characteristics.

## Methods

### Participants

This research was part of the Chinese Neuropsychological Normative (CN-NORM) project led by the Dementia Care & Research Center, Peking University Institute of Mental Health (Sixth Hospital). One hundred and nine participants were recruited between 2019 and 2021 from outpatient psychiatric or psychological departments of seven hospitals in China. The research was approved by the ethics committee at Peking University Sixth Hospital. All subjects provided written informed consent.

In the present study, the inclusion criteria for depressed elderly individuals were (1) age 55 years or older; (2) diagnosed with major depressive disorder according to DSM-5; (3) capable of understanding instructions for the cognitive tests; and (4) 30-item Geriatric Depression Scale (GDS-30) scored ≥ 10 (31). The exclusion criteria were as follows: (1) severe auditory or visual impairment; (2) a history of substance abuse or dependence; (3) comorbidity of Parkinson's disease, schizophrenia, bipolar disorder, or any other neurological or mental disorder; (4) suspected dementia diagnosed by the senior geriatric psychiatrists according to the diagnostic criteria of dementia in International Classification of Diseases and Related Health Problems 10th Revision (ICD-10); and (5) modified electric convulsion therapy (MECT) within the 6 months prior to enrollment.

After enrollment, all participants' sociodemographic characteristics, including age, sex, education level, living arrangements (living alone or with others), marital status (married or other), and medical history, were ascertained by questionnaires.

### Assessment of Depressive Symptoms

Depressive symptoms were evaluated using the Chinese version of the GDS-30 (31, 32). The GDS is widely used to measure depressive symptoms among the older adults with a yes/no response format which is less confusing and easy to apply. Each question was specially designed for elderly subjects to avoid misunderstanding and resistance. The scale has a total score ranging from 0 to 30. The GDS covers depressive symptoms including sad mood, lack of energy, agitation, worry, apathy, social withdrawal, and cognitive symptoms (33). Somatic symptoms, such as appetite and sleep disturbances, are excluded to prevent confusion (34) since somatic complaints could also be the consequence of somatic diseases, medication side effects, and the natural aging process (35). The Chinese version of the GDS-30 has proven reliable and valid in the Chinese population (34). Scores equal to or higher than ten were defined as the presence of depressive symptoms in this research.

### Cognitive Assessment

General cognitive function was evaluated by the Hong Kong Brief Cognitive test (HKBC). Developed by Chiu et al. (36) for older people with lower education, the HKBC test covers multiple cognitive domains, including immediate and delayed recall, attention, recent memory, orientation, frontal lobe function, general knowledge, visuospatial construction, executive function, and language.

A battery of comprehensive neuropsychological tests from the CN-NORM Consensus Battery (CNCB) was administered to assess memory, information processing speed, attention, executive function, language ability, and visuospatial function (37). In detail, memory was assessed by the Hopkins Verbal Learning Test-Revised (HVLT-R) and the logic memory subtest from the Wechsler Memory Scale-Revised for China (WMS-RC). The Digit Symbol Test (DST) from the Wechsler Adult Intelligence Scale-Revised for China (WAIS-RC), the Stroop Word, and the Stroop Color trial were used to assess information processing speed. Executive function was assessed by the Digit Span backward from WAIS-RC and the Stroop Color-Word trial. Language ability and attention were evaluated by the Digit Span forward and the Boston Naming Test (BNT), respectively. We used the Judgment of Line Orientation Test (JLOT) and the silhouette subtest from the Visual Object and Space Perception (VOSP) test to measure visuospatial function. All evaluations were conducted by trained investigators.

### Statistical Analysis

We performed latent class analysis using all items from the GDS-30 as indicators to investigate the latent subgroups of late-life depression. Scores of ten reverse coding items were reversed. We selected information criteria (ICs) including Bayesian information criteria (BIC), Akaike information criteria (AIC), and sample-size adjusted BIC (aBIC) in which lower values suggest better model fit. It has been reported in a study (38) that BIC performed better than other ICs. We also used the Vuong-Lo-Mendell-Rubin likelihood ratio test (LMR-LRT) and boot-strapped likelihood ratio test (BLRT) to evaluate whether a k-class model fits better than a k-1 class model. An entropy value closer to 1 indicates better classification accuracy. In selecting the final model, the number of subjects in each class was also considered for parsimony reasons.

After identifying the optimal model, we assigned each case to a specific class based on its posterior class membership probabilities. Raw scores of cognitive tests were converted into standardized *z* scores, and then the *z* scores were summed up and averaged to generate the *z* scores of the cognitive domain. Sociodemographic and clinical characteristics together with cognitive functions were compared between groups in univariate analyses using the Mann–Whitney U test, chi-square test, and *t* test. Effect sizes (Cohen's *d*s) of differences in general cognitive function and cognitive domains between groups were calculated using Psychometrica (39). Variables with statistical significance (*p* value < 0.05) were included in logistic regression analysis to explore the association between class membership and the characteristics with significant differences. Depression severity was not adjusted when applying univariate and multivariate analyses. This method was in accord with a previous data-driven study (40). We presumed that the depression severity was closely intertwined with the characteristics in the two groups, and controlling the depression severity would hamper the further comparison between groups and introduce bias. We performed the latent class analysis on Mplus version 7.4 and the other analyses on SPSS 23.

## Results

One hundred and nine LLD subjects with a mean age (SD) of 68.44 (7.04) years were recruited for the study, of whom 62.4% (*n* = 68) were female. The average GDS (SD) score was 19.76 (5.55). Among the 109 participants, four subjects had no information on living arrangements, marital status, or past medical history.

**Figure 1:**
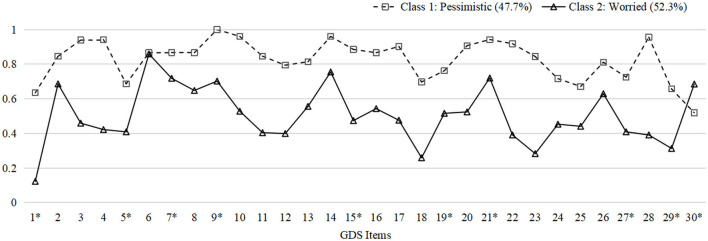
Probability plot of GDS items in two classes. The plot indicates the probabilities of endorsing every item in GDS within each latent group. Y-axis: Probabilities. X-axis: 1. Satisfied*; 2. Dropped activities and interests; 3. Empty; 4. Bored; 5. Hopeful*; 6. Bothered by thoughts; 7. Good spirits*; 8. Afraid something bad is going to happen; 9. Happy*; 10. Helpless; 11. Restless; 12. Prefer to stay at home; 13. Worry about the future; 14. Memory problems; 15. Wonderful to be alive*; 16. Downhearted; 17. Worthless; 18. Worry about the past; 19. Find life exciting*; 20. Hard to get started; 21. Full of energy*; 22. Hopeless; 23. Think most people are better off; 24. Upset; 25. Crying; 26. Have trouble concentrating; 27. Enjoy getting up*; 28. Avoid social gatherings; 29. Easy to make decisions*; 30. Clear mind* (*: Reverse coding). GDS, Geriatric Depression Scale.

Model fit information of one- to four-class models resulting from LCA are listed in Table 1. In general, the two-class model was chosen as the best fit model based on its lowest BIC, significant LMR-LRT, and BLRT, as well as interpretability. Models with more than two classes were excluded considering the insignificant LMR-LRT results and the principle of parsimony. In addition, the entropy (0.953) did not improve with a three-class model. Figure 1 illustrates the distribution of probabilities for all 30 items within two classes.

**Table 1:** Model fit information.

	**Loglikelihood**	**AIC**	**BIC**	**aBIC**	**Entropy**	**LMR-LRT**	**BLRT**	**N**
1 class	−1996.445	4052.891	4133.631	4038.835	-	-	-	109
2 classes	−1805.123	3732.247	3896.419	3703.667	0.953	0.000	0.000	52/57
3 classes	−1760.199	3704.391	3952.001	3661.293	0.953	0.244	0.000	24/30/55
4 classes	−1720.536	3687.071	4018.107	3629.443	0.970	0.446	0.000	23/30/12/44

*Parameters of latent class analysis for one to four-class models are listed. BIC, Bayesian Information Criteria; AIC, Akaike Information Criteria; aBIC, sample-size adjusted BIC; LMR-LRT, Vuong-Lo-Mendell-Rubin likelihood ratio test; BLRT, boot-strapped likelihood ratio test*.

The two-class model consisted of class 1 with 52 subjects (47.7%) and class 2 with 57 subjects (52.3%). Subjects in the two classes shared several common symptoms but differed in certain traits. Participants in class 1 were more inclined to rate items related to pessimistic thoughts, including “feel helpless,” “bored,” “empty,” “hopeless,” and “worthless.” Subjects in class 2, characterized by frequent worrying thoughts, rumination, and inability to concentrate, reported being “bothered by thoughts that cannot get out of head,” “afraid something bad is going to happen,” and “worrying about the future.” Consequently, we named class 1 as “pessimistic” and class 2 as “worried” based on the diverse patterns described above. The probabilities of endorsing every item of GDS within each group are demonstrated in Supplementary Table 1 in the Supplementary File.

**Figure 2:**
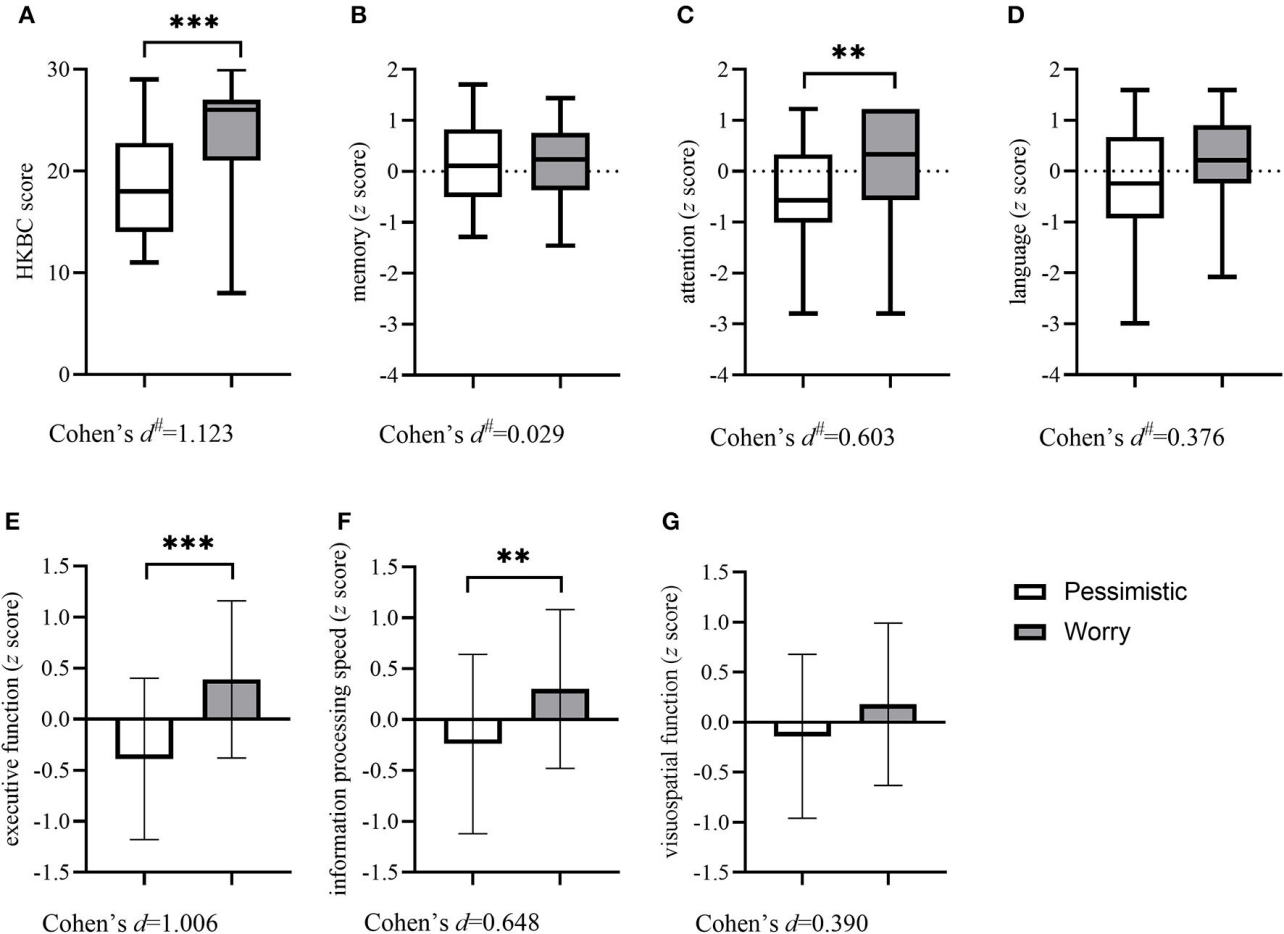
Comparisons of the HKBC test and six cognitive domains between groups. Column (with errors) and box (with min and max) plots show the differences in general cognitive function and six cognitive domains between the two groups. **(A)** HKBC; **(B)** Memory; **(C)** Attention; **(D)** Language; **(E)** Executive function; **(F)** Information processing speed; **(G)** Visuospatial function. HKBC, Hong Kong Brief Cognitive test.^#^ Effect size calculated as η2 and then transformed into Cohen's *d*. **p < 0.01, ***p < 0.001.

Table 2 shows the sociodemographic and clinical characteristics and cognitive function of the two groups. There were no significant differences in sociodemographic characteristics between the two classes. Regarding clinical characteristics, the results revealed that the subjects in the “pessimistic” group were younger at depression onset (*p* = 0.016) with a higher prevalence of past depression (*p* = 0.048) and higher GDS scores (*p* < 0.001), while the “worried” group had more physical comorbidities (*p* = 0.001) and a higher rate of past history of anxiety (*p* = 0.046). The “worried” group also presented a higher prevalence of stroke (*p* = 0.042) and hyperlipidemia (*p* = 0.002). Concerning cognitive function, the two groups differed in general cognitive function measured by the HKBC test (*p* < 0.001, Cohen's *d*: 1.123), executive function (*p* < 0.042, Cohen's *d*: 1.006), information processing speed (*p* = 0.002, Cohen's *d*: 0.648), and attention (*p* = 0.002, Cohen's *d*: 0.603), with the “worried” group performing better in the HKBC test and all three cognitive domains. The comparisons of all six cognitive domains and the HKBC test between groups, along with the effect sizes, are illustrated in Figure 2.

**Table 2:** Comparison of sociodemographic and clinical characteristics as well as cognitive function between latent classes.

	**Class 1 Pessimistic (*N* = 52)**	**Class 2 Worried (*N* = 57)**	***p* value**
**Sociodemographic characteristics**			
Age, median (IQR), years	69.09 (7.00)	68.20 (9.40)	0.701^a^
Sex, *n* (%)			0.079^b^
Male	24 (46.2%)	17 (29.8%)	
Female	28 (53.8%)	40 (70.2%)	
Education level, *n* (%)			0.087^b^
Primary school and below	8 (15.4%)	4 (7.0%)	
Secondary school	13 (25.0%)	8 (14.0%)	
High school and above	31 (59.6%)	45 (78.9%)	
Living area			0.703^b^
Rural	4 (8.2%)	3 (5.4%)	
Urban	45 (91.8%)	53 (94.6%)	
Single/divorced/widowed, *n* (%)	17 (34.7%)	15 (26.8%)	0.380^b^
Living alone, *n* (%)	7 (14.3%)	8 (14.3%)	1.000^b^
Current smoking, *n* (%)	0	4 (7.1%)	0.121^b^
Current drinking, *n* (%)	2 (4.1%)	3 (5.4%)	1.000^b^
**Clinical characteristics**			
No. physical comorbidities, median (IQR)	1(2)	2 (3)	0.001^a^
Hypertension, *n* (%)	23 (46.9%)	26 (46.4%)	0.958^b^
Stroke, *n* (%)	3 (6.1%)	11 (19.6%)	0.042^b^
Transient Ischemic Attack, *n* (%)	2 (4.1%)	6 (10.7%)	0.279^b^
Hyperlipidemia, *n* (%)	10 (20.4%)	28 (50.0%)	0.002^b^
Diabetes, *n* (%)	12 (24.5%)	8 (14.3%)	0.184^b^
Cardiac Diseases, *n* (%)	6 (12.2%)	17 (30.4%)	0.025^b^
Poor sleeping quality (self-rated), *n* (%)	37 (75.5%)	39 (69.6%)	0.502^b^
History of depression, *n* (%)	33 (67.3%)	27 (48.2%)	0.048^b^
Age of depression onset, median (IQR), years	59.00 (26)	65.50 (11)	0.016^a^
Family history of depression, *n* (%)	5 (9.6%)	2 (3.5%)	0.255^b^
History of anxiety, *n* (%)	8 (16.3%)	19 (33.9%)	0.046^b^
GDS score	24.81 ± 2.50	15.16 ± 2.91	<0.001^c^
**Cognitive function**			
HKBC, median (IQR)	18.00(9.00)	26.00 (6.00)	<0.001^a^
Executive function^*^, mean (SD)	−0.39 (0.79)	0.39 (0.77)	<0.001^c^
Information processing speed^*^, mean (SD)	−0.24 (0.88)	0.30 (0.78)	0.002^c^
Memory^*^, median (IQR)	0.11 (1.33)	0.23 (1.12)	0.903^a^
Attention^*^, median (IQR)	−0.57 (1.34)	0.33 (1.79)	0.002^a^
Visuospatial function^*^, mean (SD)	−0.14 (0.82)	0.18 (0.81)	0.055^c^
Language^*^, median (IQR)	−0.24 (1.60)	0.21 (1.14)	0.055^a^

* *, Standardized z score*.

a *, Mann–Whitney U test*.

b *, Chi-square test*.

c *, T test*.

The results (Table 3) of logistic regression analysis revealed that lower HKBC scores (OR = 1.284, 95% CI: 1.063–1.552) and lower executive function (OR = 1.285, 95% CI: 1.096–1.506) were significantly associated with the “pessimistic” group. No significant associations were observed between class membership and the number of physical comorbidities, past history of depression, age of depression onset, past history of anxiety, information processing speed, or attention ability.

**Table 3:** Logistic regression of demographic, clinical and cognitive variables on latent class membership.

	**B**	**SE**	**Sig**.	**OR, (95% CI)**
No. physical comorbidities	0.312	0.219	0.155	1.366, (0.889, 2.098)
History of depression (ref: no)	−0.687	0.854	0.421	0.503, (0.094, 2.684)
Age of depression onset	0.040	0.036	0.258	1.041, (0.971, 1.116)
History of anxiety (ref: no)	1.234	0.788	0.118	3.434, (0.733, 16.097)
HKBC	0.250	0.097	0.010	1.284, (1.063, 1.552)
Executive function^*^	0.250	0.081	0.002	1.285, (1.096, 1.506)
Information processing speed^*^	−0.099	0.065	0.125	0.905, (0.798, 1.028)
Attention^*^	−0.100	0.053	0.060	0.905, (0.816, 1.004)

*Binary logistic regression was performed to investigate the association between class membership and the following characteristics with significant differences between groups: number of physical comorbidities, history of depression and anxiety, age of depression onset, HKBC score, executive function, information processing speed, and attention*.

* *, Multiplied by 10 concerning the relatively small range of z scores*.

## Discussion

Focusing on the heterogeneity of LLD, this study applied latent class analysis to depressed older adults and further scrutinized the distinct characteristics and cognitive patterns between groups. To conclude, we discovered two discrepant groups with different symptom patterns: a “pessimistic” group with a high tendency to express pessimistic ideas and a “worried” group bothered by worry symptoms. The two groups differed in the number of physical comorbidities, past history of depression and anxiety, and cognitive function. Lower general cognitive function and executive function were associated with membership in the “pessimistic” group.

The “pessimistic” group discovered in this research is partially in accord with the results of previous LCA studies conducted in depressed older adults. Veltman et al. (28) reported a melancholic group, in which subjects were also most likely to endorse items of depressed mood, fatigue/energy loss, guilt/worthlessness, and suicidal ideation. Pérez-Belmonte et al. (29) also found a melancholic class with high probabilities of pessimism, hopelessness, and guilt. Furthermore, studies conducted in adult subjects with MDD resulted in a melancholic group with similar characteristics (41, 42). Aside from these data-driven studies, our result is also supported by Gallagher et al. (43), who found that a later age of onset was correlated with less sadness, fewer feelings of worthlessness, and less excessive guilt in older adults with MDD. Together with these results, our findings suggest that the subjects characterized by negative feelings and pessimistic thoughts might belong to a consecutive subtype of MDD both in adulthood and later-life.

On the other hand, a group with marked worry or anxiety symptoms has not been reported in previous data-driven studies targeting LLD. Worry is defined as “a chain of thoughts and images, negatively affect-laden and relatively uncontrollable” by Borkovec et al. (44), and severe worry is considered an essential part of anxiety symptoms which are highly prevalent in LLD (45). Compared with non-anxious LLD subjects, depressed older adults with comorbid anxiety showed a decreased response to antidepressants, slower remission (46), and distinct neurobiological changes (47–49). Additionally, although the literature investigating the worry symptoms in LLD is sparce, there is evidence that worry symptoms have been associated with poor efficacy of antidepressants (50) in LLD, cortex volume changes (51), and altered brain functional connectivity in late-life anxiety subjects (52) and older adults (53). Overall, these findings imply that LLD subjects with comorbid anxiety or prominent worry symptoms might be clustered into a unique subtype of LLD. More studies to are needed to confirm this particularity.

Second, in terms of cognitive function, our study found that the “pessimistic” group with a lower mean age of depression onset was more impaired in executive function, information processing speed, and attention. Conflicting with several previous studies that reported severer executive dysfunction in LOD subjects (16, 17, 54), our result is partially comparable to one prior study showing more executive impairment in EOD subjects (19), and implies the potential negative effect of the longer exposure to depression on the prefrontal cortex. A systemic review also revealed that melancholic patients had more disturbances in executive function and processing speed (55). However, inconsistency is understandable because depression severity was not controlled between groups, which also affected cognitive performance (56). This finding might be attributed to negative self-referential thinking in the “pessimistic” group, which leads to an impaired ability to disengage from negative internal thoughts and a failure to shift attention toward external cognitive stimuli (57), and eventually giving rise to deficits in working memory and executive dysfunction (58).

In contrast, the “worried” group showed relatively less impaired cognitive function, which is supported by several studies showing no negative effect of anxiety on cognitive functions (59, 60). The Yerkes-Dodson law (61) proposed an inverted u-shaped relationship between the state of stress and cognitive performance, in which a proper amount of stress or arousal contributed to better cognitive function. In addition, Beaudreau et al. (62), who found that worry attenuated the negative effect of depressive and anxiety symptoms on inhibitory control, suggested that the personality trait of perfectionism characterized by higher motivation for better performance and worry level could be a possible explanation. Moreover, it has been hypothesized that abnormalities in the functional connectivity between and within the executive control network (ECN) and the default mode network (DMN) (63, 64) together with the failure of DMN deactivation during cognitive tasks (65) lead to negative cognitive bias and cognitive impairment in LLD subjects (66). Brain imaging evidence also indicated a correlation between worry and the abnormalities of limbic-prefrontal functional connectivity as well as intra and inter-DMN connectivities (52, 53). Based on these discoveries, we presume that these two groups may have distinct patterns of neurobiological alterations.

Finally, we found that lower general cognitive function and executive function were associated more often with the membership of the “pessimistic” group, which also has clinical implications. It has been shown in several studies that executive dysfunction is related to poor or slow response to antidepressants, higher functional disability (67), and dementia conversion (68). Hence, we suggest that it is valuable to regularly evaluate and monitor the cognitive function of LLD subjects, especially those with pessimistic characteristics, which would help clinicians determine whether intensive antidepressant treatment and compensatory cognitive interventions are necessary to achieve better prognoses.

Our findings indicated that the heterogeneity of late-life depression could be embodied in diverse sets of symptoms and corresponding patterns of cognitive impairment. We speculate that although the age of depression onset or clinical specifiers may contribute to this heterogeneity in part, the major section still remains undiscovered, similar to the “iceberg theory,” which could explain why some studies have failed to find significant discrepancies (69). Future studies with data-driven and biological approaches are encouraged to confirm the existing subtypes and to uncover more convincing phenotypes of late-life depression.

The present research has several limitations. First, we were not able to exclude the potential effect of severity of the depressive symptoms in the analyses, since the GDS scores were used as the indicators to generate the latent classes. This might be improved by introducing other assessment instruments such as Hamilton Depression Rating Scale (HDRS) and controlling the scores in the analyses. However, the findings in our study may imply that the different levels of depression severity of the two groups could be their characteristics in real-world clinical situation. Second, although the GDS-30 consists of factors such as “worry” and “apathy,” specialized methods to evaluate these symptoms, such as the Hamilton Anxiety Rating Scale (HAMA) or Apathy Evaluation Scale (AES), were not included in this study, which could have better described the discrepancies in symptom profiles between groups. Third, the diagnosis was based on psychiatric interviews, rather than the structured diagnostic tools such as the Mini International Neuropsychiatric Interview (MINI). Also, the medical records on the number of depressive episodes, and the use of psychotropics or benzodiazepines were not retrievable in the study. The profiles of clinical characteristics of the two subtypes might not be investigated thoroughly. Additionally, our study did not include older adults without depression as normal controls. The comparison between the two latent groups and the normal control could have provided us with more information about the cognitive profiles of the two groups. Moreover, the stability and transition of the heterogeneity of LLD as well as the cognitive changes over time were not considered in our study. Recently Veltman et al. (70) conducted latent transition analysis to examine the stability of latent groups over time, and they found that although the latent groups remained relatively stable, physiological aging seemed to blur the differentiation between latent groups, suggesting that the effect of the aging process should also be considered in future studies of LLD heterogeneity. Last but not least, the small sample size might have hindered generalizability of the results to the general population, because small numbers of subjects with distinct characteristics might not be discovered when applying LCA. Also, the subjects in our study had moderate or high-level education. It might limit the generalizability of the present findings. However, our research confirmed some results from previous studies and underlined the cognitive difference between latent groups and thus might be viewed as part of the preliminary foundation for advanced studies in the future. Further studies are warranted to include separate samples with different sociodemographic characteristics to validate the heterogeneity of late-life depression.

## Conclusion

To conclude, this study identified two subgroups of LLD subjects: one “pessimistic” group characterized by a high prevalence of negative and pessimistic thoughts, and the other “worried” group with relatively high probability of exhibiting worry symptoms. The two groups differed in specific clinical characteristics and cognitive functions. This study provides a deeper insight that heterogeneous subgroups in LLD also differ in cognitive profiles, which may shed new light on further research of the heterogeneity of LLD. The symptoms of pessimistic thinking and worry of LLD could be a neoteric starting point to better delineate the heterogeneous patterns of LLD.

## Data Availability Statement

The dataset generated and analyzed during the current study are not publicly available because we are preparing additional manuscripts. However, they are available upon the reasonable request to the corresponding authors.

## Ethics Statement

The studies involving human participants were reviewed and approved by Peking University Sixth Hospital. The patients/participants provided their written informed consent to participate in this study.

## Author Contributions

L-QW conducted the study design and statistical analysis and drafted the original manuscript. L-QW, Z-LF, L-HT, MZ, T-HZ, WD, SL, H-NW, NZ, Q-YM, H-ZL, L-CW, Y-NZ, and YZ participated in the data collection. HW and XY contributed to the study's design, statistical analysis, data interpretation, and revised and reviewed the article. All authors contributed to the article and approved the submitted version.

## Funding

This work was supported by the National Key Research and Development Program of China (2018YFC1314200 and 2017YFC1311100). The funding source had no role in the study design, data collection, analysis and interpretation, or decision to submit the manuscript for publication.

## Conflict of Interest

The authors declare that the research was conducted in the absence of any commercial or financial relationships that could be construed as a potential conflict of interest.

## Publisher's Note

All claims expressed in this article are solely those of the authors and do not necessarily represent those of their affiliated organizations, or those of the publisher, the editors and the reviewers. Any product that may be evaluated in this article, or claim that may be made by its manufacturer, is not guaranteed or endorsed by the publisher.
