# Supplementary material for: Heterogenous Subtypes of Late-Life Depression and Their Cognitive Patterns: A Latent Class Analysis
Source: Front Psychiatry. 2022 Jul 6;13:917111. doi: 10.3389/fpsyt.2022.917111 (PMC9298648; doi:10.3389/fpsyt.2022.917111)
Supplement: Supplementary file 1 [file Table_1.DOCX]

**Supplemental materials**

Table S1. GDS items with top 10 highest probabilities in each class.

| Items | Probability (Class 1) | Items | Probability (Class 2) |
| --- | --- | --- | --- |
| 9^*^. Happy most of the time | 1.000 | 6. Bothered by thoughts cannot get out of head | 0.859 |
| 10. Feel helpless | 0.960 | 14. Have problems with memory | 0.755 |
| 14. Have problems with memory | 0.960 | 21^*^. Feel full of energy | 0.720 |
| 28. Avoid social gatherings | 0.956 | 7^*.^ In good spirits | 0.718 |
| 4. Often get bored | 0.942 | 9^*.^ Happy most of the time | 0.702 |
| 21^*^. Feel full of energy | 0.942 | 2. Dropped activities and interests | 0.686 |
| 3. Life is empty | 0.939 | 30^*^. Mind as clear as it used to be | 0.685 |
| 22. Feel hopeless | 0.918 | 8. Afraid something bad is going to happen | 0.648 |
| 20. Hard to get started on new projects | 0.907 | 26. Have trouble concentrating | 0.629 |
| 17. Feel worthless | 0.902 | 13. Worry about the future | 0.556 |
| 15^*^. Wonderful to be alive | 0.885 | 16. Downhearted and blue | 0.543 |
| 7^*^. In good spirits | 0.867 | 10. Feel helpless | 0.528 |
| 6. Bothered by thoughts cannot get out of head | 0.866 | 20. Hard to get started on new projects | 0.524 |
| 8. Afraid something bad is going to happen | 0.866 | 19^*^. Find life exciting | 0.516 |
| 16. Downhearted and blue | 0.866 | 17. Feel worthless | 0.476 |
| 11. Restless and fidgety | 0.846 | 15^*^. Wonderful to be alive | 0.474 |
| 2. Dropped activities and interests | 0.845 | 3. Life is empty | 0.459 |
| 23. Think most people are better off | 0.844 | 24. Frequently get upset over little things | 0.453 |
| 13. Worry about the future | 0.814 | 25. Frequently feel like crying | 0.441 |
| 26. Have trouble concentrating | 0.811 | 4. Often get bored | 0.422 |
| 12. Prefer to stay at home | 0.794 | 5^*^. Hopeful about future | 0.409 |
| 19^*^. Find life exciting | 0.762 | 27^*^. Enjoy getting up in the morning | 0.409 |
| 27^*^. Enjoy getting up in the morning | 0.725 | 11. Restless and fidgety | 0.404 |
| 24. Frequently get upset over little things | 0.716 | 12. Prefer to stay at home | 0.399 |
| 18. Worry about the past | 0.697 | 22. Feel hopeless | 0.391 |
| 5^*^. Hopeful about future | 0.686 | 28. Avoid social gatherings | 0.391 |
| 25. Frequently feel like crying | 0.671 | 29^*^. Easy to make decision | 0.313 |
| 29^*^. Easy to make decision | 0.657 | 23. Think most people are better off | 0.283 |
| 1^*^. Satisfied with life | 0.635 | 18. Worry about the past | 0.259 |
| 30^*^. Mind as clear as it used to be | 0.519 | 1^*^. Satisfied with life | 0.123 |

The probabilities of endorsing every item of GDS within each group are listed in the table. ^*^: Reverse coding items.
